# Ovarian teratoma-associated anti-NMDAR encephalitis: a systematic review of reported cases

**DOI:** 10.1186/s13023-014-0157-x

**Published:** 2014-10-14

**Authors:** Pedro Acién, Maribel Acién, Eva Ruiz-Maciá, Carlos Martín-Estefanía

**Affiliations:** Obstetrics and Gynecology Service, San Juan University Hospital, Alicante, Spain; Neurology, San Juan University Hospital, Alicante, Spain; Department/Division of Gynecology, Miguel Hernández University, San Juan Campus, 03550 Alicante, Spain

**Keywords:** Anti-NMDAR encephalitis, Anti-NMDA-Receptor antibodies, Dermoid cyst, Limbic encephalitis, Ovarian teratoma

## Abstract

The association of ovarian teratoma and anti-N-methyl-D-aspartate receptor (NMDAR) encephalitis is a serious and potentially fatal pathology that occurs in young women and that is under-recognized. Our objectives were to analyze prevalence and outcome of this association, and increase awareness over this pathology. MEDLINE and SCOPUS for all studies published prior to November 30, 2013 including the search terms: “encephalitis” and “teratoma” were considered. All articles (119) reporting one or more cases of anti-NMDAR encephalitis and confirmed ovarian teratoma (174 cases) were included. No language restrictions were applied. Suspicious cases with no evidence of ovarian teratoma (n = 40) and another type of encephalitis also associated to ovarian teratoma (n = 20) were also considered for comparison and discussion. Data of publication and case report, surgery and outcome were collected. The distribution of published cases is heterogeneous among different countries and continents, probably in relation with level of development and health care. The mean patient age is 24 years and in the majority of cases (74%), a mature teratoma was identified, sometimes microscopically following ovarian removal or at autopsy. The clinical presentation featured psychiatric symptoms and behavioural changes, with a median delay for surgery of 28 days. Twelve women died (7%), most frequently from encephalitis-related complications. In conclusion, the association ovarian teratoma and anti-NMDAR encephalitis is relatively unknown or not reported in many countries and among gynecologists. Heightened recognition of behavioral changes, diagnosis through transvaginal ultrasound and subsequent tumor removal in addition to diagnostic confirmation through the presence of anti-NMDAR antibodies must be emphasized.

**Electronic supplementary material:**

The online version of this article (doi:10.1186/s13023-014-0157-x) contains supplementary material, which is available to authorized users.

## Additional non-English language abstract (Spanish)

La asociación de teratoma ovárico y encefalitis anti-NMDAR es una patología grave y potencialmente mortal, poco conocida y que ocurre en mujeres jóvenes. El objetivo de esta revisión sistemática fue analizar la prevalencia y el devenir de esta asociación, así como llamar la atención sobre el necesario conocimiento de esta patología. Fueron considerados todos los estudios encontrados en MEDLINE y SCOPUS, empleando los términos de búsqueda “encephalitis” y “teratoma”, hasta el 30 de Noviembre de 2013; y fueron incluidos en la revisión todos los artículos (119) que reportaban uno o mas casos de encefalitis anti-N-methyl-D-aspartate receptor (NMDAR) con teratoma ovárico confirmado (174 casos). No hubo restricciones por idioma. Los casos sospechosos pero sin demostrar el teratoma ovárico (n = 40), así como otros tipos de encefalitis también asociados a teratoma (n = 20), fueron igualmente considerados para comparación y discusión. Se recogieron los datos de la publicación y del “case report”, incluyendo manifestaciones clínicas, tratamiento, cirugía practicada, resultados y devenir de la paciente a corto y largo plazo. El análisis de resultados muestra que la distribución de los casos es muy heterogénea entre los diferentes países y continentes, probablemente en relación con el nivel de desarrollo y los cuidados de salud. La edad media de las pacientes fue de 24 años, y en la mayoría de los casos (74%) se identificó teratoma maduro (quiste dermoide), siendo algunas veces microscópico o un hallazgo en la autopsia. La presentación clínica fue generalmente con síntomas psiquiátricos y cambios del comportamiento. El retraso medio para un correcto diagnóstico y cirugía fue de 28 días, falleciendo finalmente 12 mujeres (7%), especiamente por complicaciones relacionadas con la encefalitis. En conclusión, la asociación de teratoma ovárico y anti-NMDAR encefalitis es poco conocida o no reportada en muchos paises y entre ginecólogos, debiendo ponerse atención y ser enfatizados los cambios comportamentales, el diagnóstico mediante ecografía transvaginal, la pronta exéresis tumoral y la confirmación diagnóstica mediante la positividad de los anticuerpos anti-NMDAR.

## Introduction

Limbic encephalitis was initially described as a paraneoplastic syndrome characterized by the rapid development of confusion and personality changes, irritability, depression, seizures, short-term memory loss, sometimes dementia and high MRI T2 and FLAIR signal intensity involving one or both medial temporal lobes [1]. It was primarily associated with lung or testicular cancer and with antibodies against intracellular neuronal antigens [2]. However, recently a new type of encephalitis was described in women with ovarian teratoma and antibodies that react with neuronal cell surface auto-antigens [3,4]. Hence, encephalitis, considering those related to paraneoplastic syndromes and their associated antibodies [5], could be classified according to the auto-antibody that causes the disease, such as anti-Hu, which is associated with small-cell carcinoma of the lung; anti-Ma2, which is associated with germ-cell tumors of the testis and anti-NMDAR (*N*-methyl-D-aspartate receptor), which is associated with tumors of the ovary.

In fact, Vitaliani et al. [3] and Dalmau et al. [4] described a disorder that appeared to represent a new subcategory of severe and potentially lethal, yet treatment-responsive, paraneoplastic encephalitis, which they described as “paraneoplastic anti-N-methyl-D-aspartate receptor encephalitis associated with ovarian teratoma”. The affected patients were women who developed prominent psychiatric symptoms, seizures, memory deficits and a decreased level of consciousness that often required ventilatory support. Three salient features of the syndrome included the young patient age, the association with ovarian teratoma and the detection of antibodies to unknown antigens that were predominantly expressed in the cell membranes of hippocampal neurons (neuropil antigens) [6].

All of the studies described above were performed by a group headed by J. Dalmau (Department of Neurology, University of Pennsylvania, PA, USA) that reviewed previously reported cases in 2007, along with eight additional patients in whom they identified the target autoantigens as heteromers containing NR1 and NR2 subunits of the NMDA receptor that were expressed by the associated tumors [4]. This syndrome is now referred to as anti-NMDAR encephalitis, and although it was initially associated with ovarian teratoma in women, it has now been described in men and is increasingly being recognized in children [7,8]. However, gynecologists may not be aware of this serious and potentially fatal pathologic condition that frequently originates in the ovary.

## Objectives

This review discusses the incidence and prevalence, country of origin, histological type of ovarian teratoma and outcome of the teratoma-associated anti-NMDAR encephalitis and provides practical guidance for the general gynecologist, thus contributing to the diffusion of knowledge regarding this association that, notwithstanding its clinical infrequency, has the potential for severe consequences.

## Methods for review

### Sources

A comprehensive search of PubMed and SCOPUS was performed for all studies published prior to November 30, 2013, using the search terms “encephalitis” and “teratoma”, which yielded 192 articles in PubMed and 274 in SCOPUS. A systematic review of these papers was performed, and after removal of repeated articles from both searches and cases involving male patients, the titles and abstracts of all articles were evaluated to determine whether case reports with ovarian teratoma were included. There were no language restrictions, although for articles in Chinese or Japanese, only abstracts were consulted.

### Study selection

All publications reporting one or more cases with encephalitis and ovarian teratoma, including suspicious cases in which the tumor had not been found, were selected for review and full publication analysis. Altogether, 119 reports containing a total of 173 cases of anti-NMDAR encephalitis and ovarian teratoma were identified from 1997 until November 30, 2013. To these, we have added our case attended in 2012–2013 and recently published [9], making a total of 174 cases of ovarian teratoma-associated anti-NMDAR encephalitis.

**Figure 1:**
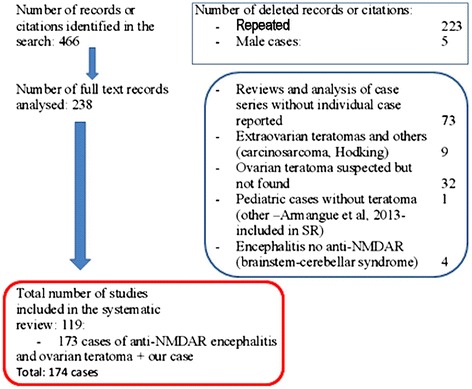
**Flow diagram for the systematic review.**

Moreover, 7 of the aforementioned papers and another 32 papers published between 2009 and 2013 described 40 additional cases of anti-NMDAR encephalitis and suspicious of teratoma in which, following ultrasound, CT or MR, an ovarian teratoma had not been found. Other 11 pediatric cases (seven in females) of anti-NMDAR encephalitis without teratoma [8], and 20 female cases of ovarian teratoma with brainstem-cerebellar manifestation with or without opsoclonus-myoclonus syndrome reported by Armangue et al. [10] (including three cases previously reported [11-13]) have also been analyzed or independently commented on. Flow diagram for the study is shown in Figure 1 as supplemental data.

The following data were collected from all of the articles containing any case of anti-NMDAR encephalitis and ovarian teratoma: authors, journal and year of publication, country of birth or hospital service where the case was studied, number of cases included with or without ovarian tumor, patient age, clinical presentation, type of ovarian teratoma, tumor size and laterality, medical treatment, time to surgery, surgery performed, outcome and time to recovery. These data were tabulated in a *Supplemental Data File* (Additional file 1: Table S1), and were also entered into SPSS statistics (IBM, Spain) and statistically analyzed. Times to discharge, partial recovery or full recovery were considered following the case reported if it was mentioned.

We also analysed the case series of anti-NMDAR encephalitis, with or without teratoma, in which the cases were not individually reported, and the most comprehensive reviews of paraneoplastic limbic encephalitis for discussion.

### Statistical analysis

Data were introduced in a SPSS-15 file being statistically analyzed. Percentages, mean and standard deviation, and median with minimal and maximal values were obtained. Comparison of independent means (Student’s T-test), comparison of two proportions (relative risk –RR-), a 95% confidence interval (CI) and the chi-squared test were used to compare the groups. RSigma (Systat Software, San Jose, California, USA) and PEDro (Physiotherapy Evidence Database, Sidney, Australia) software were also used. All p values reported are 2 tailed and p value of 0.05 or less was considered significant.

## Results

174 cases of anti-NMDAR encephalitis caused by ovarian teratoma have been studied and analyzed (see Additional file 1: Table S1 at Supplementary Data).

The publication years for cases of anti-NMDAR encephalitis and ovarian teratoma are presented in Table 1A. It is notable that the number of papers and case reports has progressively increased since the 2007 publication of Dalmau et al. [4]. Most articles containing case reports have been published in neurology or psychiatry journals, with only 11% of studies published in gynecology journals, including those in gynecologic oncology (Table 1B). With regard to the country of birth (or eventually, the country of study), as shown in Table 1C, a global imbalance exists that can likely be attributed, at least in part, to differences in the level of development and health care. Except for a Mauritanian patient who was attended in France [14], not a single case has been reported from Africa. In Asia, only one case each has been published from China and India, whereas Japan has reported 38 cases. No cases have been reported from Russia. In the Americas, three cases have been reported from Central and South America, whereas the USA has contributed 47 cases. In addition, 14 cases have been described in papers from Spain and the USA by the Dalmau group, without specification of individual case origin [15,16].

**Table 1:** **Year of publication, journal and country of birth or study of patients with anti-NMDAR encephalitis and ovarian teratoma**

**1A. Year of publication (number of papers)**	**Number of cases**	**%**
Before/or in 2006 (13)	16	9,2
2007 (6)	14	8,1
2008 (11)	18	10,4
2009 (14)	17	9,8
2010 (18)	21	12,1
2011 (21)	27	15,6
2012 (18)	27	15,6
2013 (November 30) (18)	33	19,1
TOTAL (119)	173	100
**1B. Journal of publication**	**Number of cases**	**%**
1. Neurology or Psychiatry	86	49,7
2. Internal or general medicine	34	19,6
3. Other Specialties	34	19,6
4. Gynecological, included Oncological Gynecology	19	11,0
TOTAL	173	100
**1C. Country of birth or study**	**Number of cases**	**%**
**Africa**: (Mauritania, France)	1	0,6
**Asia**: (*Japan-38*; Taiwan-4; Singapore-3, Hong-Kong-2; and 1 in China, India, S-Korea and Lebanon)	51	29,5
**Australia**: (Australia-9; New Zeland-4)	13	7,5
**Europe**: (Spain-7; UK-7; France-6; Germany-5; 3 in Netherlands, Belgium, Italy and Turkey; and 1 in Ireland and Greece)	39	22,5
**North-America**: (*USA-47*; Canada-4, Mexico-1)	52	30,1
**Center and South-America**: (1 in Brazil, Colombia and Jamaica)	3	1,7
Spain (Barcelona) v USA (Pennsylvania)	14	8,1
TOTAL	173	100

In Table 2, the mean age (with standard deviation and median), tumor laterality, size and histological type are presented. Most published cases with anti-NMDAR encephalitis were associated with mature teratomas (dermoid cysts) (57% overall, or 74% in the subgroup of cases where the histologic type was specified), and 29 (16.7% overall, or 21.6% among the cases where histological type was specified) were immature teratomas. Certain cases manifested both types of teratoma or demonstrated immature teratoma foci (3.4%); however, in 40 cases (23%) the histological type was not reported. The mean age was similar in all groups with teratoma (23.9 ± 7.8 years; median 24 years) but was significantly lower in cases where a teratoma was not found (19.1 ± 9.8 years; median 17 years). In 20 cases of ovarian teratoma with brainstem-cerebellar manifestation with or without opsoclonus-myoclonus syndrome (a novel treatment-responsive encephalitis negative for anti-NMDAR antibodies [10]), the median age was 28.5 years; among them, there was only one case of immature teratoma and in 13 cases of mature teratoma. In the pediatric series of anti-NMDAR encephalitis without teratoma, the mean age was 6.9 years (range 2.5–12.9 years).

**Table 2:** **Age of patients, histological type of ovarian teratoma, laterality of the affected ovary and tumor size**

**Variable**	**Histological type of ovarian teratoma**				**Total of cases with teratoma**	***Ovarian teratoma not found***
	**Mature**	**Immature**	**Mature and immature**	**Not specified**		
**Age** (N)	(99)	(29)	(6)	(40)	(174)	(40)
Mean ± SD	24,1 ± 7,3	24,9 ± 8,4	26,0 ± 10,0	22,6 ± 8,4	23,9 ± 7,8^*^	19,1 ± 9,8^*^
Median(min-max)	24(7–42)	25(9–41)	26(15–39)	20(11–54)	24(7–54)	17(5–57)
**Tumor location**						
Right ovary	34(60,7)	13(23,2)	2(3,6)	7(12,5)	56	-
Left ovary	34(73,9)	11(23,9)	0-	1(2,2)	46	-
Bilateral	10(50,0)	2(10,0)	3(15,0)	5(25,0)	20	-
Not marked	21(40,4)	3(5,8)	1(1,9)	27(51,9)	52	-
Total	99(56,9)	29(16,7)	6(3,4)	40(23,0)	174	40
**Teratoma size**(cm)						
Mean ± SD(N)	5,7 ± 5,7(61) ^×^	9,0 ± 5,0(20)	11,2 ± 5,9(5)	-	6,7 ± 5,7(87)	0
Median(m-m)	4(0,1-22)	7,9(2,5-22)	11(5–20)	-	5(0,1-22)	0

^*^, p < 0,001 versus cases with teratoma (CI 1,96-7,64).

^×^, p < 0,05 versus immature and mature and immature (CI 1,03-6,37).

The tumor was located more frequently on the right ovary, but differences were not significative. Immature teratomas or mature teratomas with immature foci were larger (9–11 cm) than dermoid cysts, although it should be noted that in some cases, the teratoma was microscopic and was only found following oophorectomy [17] or at autopsy [18] several months after admission.

The clinical presentation in the majority of cases was similar to our case [9], particularly with regard to the memory impairment, psychiatric symptoms and behavioral changes, and also included psychosis, abnormal movements of the face, mouth and tongue, fever, headache, hallucinations, rhinorrhea, hypersalivation, seizures, catatonia and status epilepticus. Two affected patients also presented during pregnancy [19]. An important finding in this systematic review was the extended time lapse between hospitalization and diagnosis or surgical removal of the tumor. Many patients had been admitted to different hospitals, often psychiatric, or departments other than gynecology prior to undergoing surgery. The median time to surgery was 28 days, with a range between 2 and 455 days. Because in most cases it was not suspected that the encephalitis was caused by teratoma, the mean time to surgery was 71.4 ± 88.5 days in cases of mature teratomas and significantly shorter in cases of immature teratoma (39.4 ± 28.6 days) (Table 3).

**Table 3:** **Time to diagnosis and surgery (days) and surgery performed according to teratoma histological type**

**Histological type (N)**	**Time to diagnosis/surgery (N), median mean ± SD**	**Surgery performed**					**Autopsy N (%)**
		**Tumor excision N (%)**	**Unilateral adnexectomy N (%)**	**Bilateral cystectomy N (%)**	**Bilat. Ooph. or radical N (%)**	**No surgery N (%)**	
Mature (99)	(85), 39 days 71,4 ± 88,5^*^	54(54,5)^+^	34(34,3)	5(5,1)	2(2,0)	1(1,0)	3(3,1)
Immature (29)	(26), 27,5 days 39,4 ± 28,6^*^	8(27,6)	17(58,6)^++^	0-	4(13,8)	0-	0-
Mature + Immature (6)	(3), 14 days 14,0 ± 0,0	0-	2(33,3)	0-	3(50,0)	1(16,7)	0-
Not specified (40)	(10), 13,5 days 37,3 ± 42,5	25(62,5)	5(12,5)	1(2,5)	3(7,5)	3(7,5)	3(7,5)
Total (174)	(124), 28(3–455) days 60,6 ± 77,0	87(50,0)	58(33,3)	6(3,4)	12(6,8)	5(2,9)	6(3,4)

(N), n° of cases in which this variable is known;

*, T test, p < 0,02 (CI 3–67); ^+^, Chi2 test, p < 0,05, RR = 1,97 (CI 1,1-3,6); ^++^, RR = 1,7 (1,1-2,57).

The most frequent surgical procedure for mature teratoma removal was simple tumor excision while uni/bilateral salpingo-oophorectomy and radical surgery was reserved for patients with immature teratomas. A few isolated cases achieved recovery without tumor removal, whereas in 6 women (3.4%), the tumor was found at autopsy. Cases of recurrent dermoid cyst and recurrent encephalitis have been reported [20], whereas other patients have presented with encephalitis months or years following removal of the ovarian teratoma [21]. In contrast, the tumor was only found after a period of slow recovery from encephalitis [22] in some patients who also had negative test results in earlier diagnostic methods.

The diagnostic workup based on the definitive determination of anti-NMDAR antibodies in blood or preferably in cerebrospinal fluid (CSF) [23], and the imaging of ovarian teratoma were similar to those in our case [9]: abdomino-pelvic CT image and transvaginal ultrasound (TVU). The medical treatments were also similar and included acyclovir, methylprednisolone, ivIg, anticonvulsants and chemotherapy in cases of immature teratoma. Other effective therapies included plasmapheresis and antiepileptic and anticonvulsive therapy, with some cases requiring tracheostomy or gastrostomy.

The patient outcomes according to the teratoma histological type are shown in Table 4. A full recovery was achieved by 88% of women with mature teratoma and 76% of women with immature teratoma after several months or years. The mean time to acceptable recovery and hospital discharge was 3.6 ± 3.3 months (median 3 months; range 0.5–24 months). Permanent sequelae were observed in 11.5% of patients, and 12 women expired (7%) from encephalitis-related complications that did not depend on the histological type of teratoma.

**Table 4:** **Outcome of anti-NMDAR encephalitis patients with or without ovarian teratoma**

**Histological type of teratoma**	**OUTCOME**			**Time to discharge or partial recovery (N), median (m-m) m ± SD (months)**
	**Recovery N (%)**	**Partial recovery N (%)**	**Died N (%)**	
Mature (99)	87(87,9)	8(8,1)	4(4,0)	(47), 3(0,5-12), 3,1 ± 2,0
Immature (29)	22(75,9)	5(17,2)	2(6,9)	(9), 3,5(0,7-11), 4,3 ± 3,4
Mature + Immature (6)	4(66,7)	1(16,7)	1(16,7)	(3), 4(3–6), 4,3 ± 1,5
Not specified (40)	29(72,5)	6(15,0)	5(12,5)	(11), 3(1–24), 5,0 ± 6,4
**Total with teratoma (174)**	142(81,6)	20(11,5)	12(6,9)	(70), 3(0,5-24), 3,6 ± 3,2
Ovarian teratoma not found (40)	34(85)*	3(7,5)	1(2,5)	(10), 5,5(1–14), 5,7 ± 3,7^+^

(N), n° of cases in which this variable is known; *, not documented in two cases.

^+^, CI 1,0-4,25 versus mature teratoma.

In the 40 women with anti-NMDAR encephalitis in whom an ovarian teratoma was not found, 85% achieved a full recovery however, the time to recovery was longer (6.3 ± 4.1 months; median 5.5 months; range 1–14 months) than in patients whose ovarian teratomas were removed. In addition, three patients (7.5%) achieved only a partial recovery, and one patient died [24]. Similarly, only 50% of pediatric patients without teratoma experienced a full recovery [8], and two patients had severe residual sequelae. Among the patients with brainstem-cerebellar syndrome and teratoma [10], 74% experienced a full recovery, 16% had partial improvement, and two patients had no improvement (one of whom did not undergo surgery).

## Comment

### Strengths and weaknesses

We have included in the systematic review all detailed cases of ovarian teratoma-associated anti-NMDAR encephalitis appearing in PubMed and/or Scopus and whose data had been reported as individual case report. Observational cohorts’ studies without individual description of cases have not been included in the analysis of cases.

### Weaknesses

1. We suspect that this pathological association is underdiagnosed and infrareported. Moreover, the case has not been analyzed if the publishing journal is not in PubMed or Scopus. 2. Teratoma is not always found, especially among girls and adolescents. 3. There may be extraovarian teratoma. 4. The ovarian teratoma may also cause a novel type of encephalitis negative for anti-NMDAR, with brainstem-cerebellar manifestation and with or without opsoclonus-myoclonus syndrome [10].

### Main findings

The association of anti-NMDAR encephalitis and ovarian teratoma is a severe, prolonged and potentially fatal pathologic condition of young women that has been increasingly reported in developed countries since its identification and publication by Dalmau et al. [4]. But is uncommon in girls ≤14 years of age [25], Only 4 females younger than 12 years −6%- had a tumor in Titulaer et al. [26] study.

The most frequent clinical presentations are neuropsychiatric symptoms (especially, behavioural changes), although the neuropsychiatric manifestations may often be preceded by nonspecific prodromal symptoms. Moreover, the typical presentation of the cases include memory impairment and, later but very evocative, abnormal movements of the face, mouth and tongue and symptoms of dysautonomia. General practitioners and gynecologists should be cognizant of available imaging modalities that may be employed in the early stage and of the need for the eventual discovery of a dermoid cyst because early tumor resection with immunotherapy will facilitate patient recovery. It should also be noted that at the onset of symptoms, the results of anti-NMDA antibody testing may be negative, even in CSF [21]. Nevertheless, it has been suggested that in some patients with anti-NMDAR encephalitis the antibodies were detectable only in the CSF [27]; and Gresa-Arribas et al. [23] have reported that the sensitivity of NMDA receptor antibody testing is higher in CSF than in serum, that the titres were higher in patients with poor outcome and that antibody titres can complement clinical assessment.

### Interpretation and alternative explanations for observed results

Even small ovarian teratomas containing nervous tissue may express NMDAR subunits that react with a patient’s antibodies, thereby triggering a cascade of symptoms that is recognized as anti-NMDAR encephalitis and that mainly affects the hippocampus/forebrain regions. In such cases, tumor resection and immunotherapy result in improvement or full recovery. However, the diagnosis is difficult and time-consuming, and patients may succumb to neurological deterioration, thus necessitating a high index of suspicion and prompt intervention, even in the absence of confirmatory studies.

From this systematic review, it is apparent that awareness of the association of ovarian teratoma (usually benign or dermoid cyst) and anti-NMDAR encephalitis is low in many parts of the world, possibly because practitioners may not entertain such a diagnosis, thus resulting in a low rate of diagnosis even among affected patients. Some women may be transferred from one hospital to another or from one service to another, which may contribute to the fatality rate of approximately 7%, with the teratoma occasionally found only at autopsy. Therefore, heightened awareness will benefit patients through a more rapid identification of this association, especially in Africa, Asia and Central and South America, and particularly among gynecologists. Few reported cases have emerged from these regions, possibly because of their respective levels of health care and socio-economic development.

However, from the point of view of etnicity, Titulaer et al. [26] in a cohort multi-institutional observational study of 577 anti-NMDAR encephalitis patients have observed that Asian and African-American patients were more likely to have a teratoma (45% and 48% respectively) than Caucasian (31%) or Hispanics (27%), being p < 0.007.

On the other hand, Tachivana et al. [28] noted that in the vast majority of encephalitis cases, patients have a prodromal infection before the onset of the disease. Should this infection affect either the normal ovary or ovarian teratoma, it may trigger the expression of NMDAR-related epitopes in oocytes and lead to the development of limbic encephalitis disease [29]. Indeed, ovarian teratomas that contain large amounts of neural tissue may present an increased risk for the development of this type of life-threatening encephalitis. Subsequently, the diagnosis can be established by identification of anti-NMDA antibodies in the serum and CSF. When these antibodies are positive, even in the absence of teratoma, it has been suggested that proceeding to exploratory laparotomy may increase the possibility of identifying microscopic teratoma and may improve the outcome in patients who are refractory to immunotherapy [30], however it is unclear whether this procedure is the most appropriate.

### Generalization of the conclusions

It now appears relevant to raise the questions posed to gynecologists by the 2009 report of Kort et al. [31].

### Incidence and prevalence of this condition

Only eight cases with paraneoplastic limbic encephalitis and teratoma, some of which were recurrent [20], were described before 2005, the year in which Dalmau et al. introduced the determination of anti-NMDAR antibodies in the serum and CSF [3,6,32]. Since 2007, the year in which Dalmau et al. published their work in the Annals of Neurology [4], there has been a progressive increase in the number of articles and case reports regarding this entity. Thus, it appears that the incidence of this condition is higher than previously estimated and will continue to increase with improvements in health care, knowledge of the condition and improved socio-economic development, especially in many of the larger countries of Asia, Africa and South-America where cases have not yet been reported. General practitioners and gynecologists should be aware that the underlying cause of anti-NMDAR encephalitis may involve a simple ovarian dermoid cyst.

### Clinical presentation

Neuropsychiatric symptoms, rapid neurological deterioration, ICU admission and ventilator support are often initiated before the diagnosis is established. Patients often require long periods of support, and they may also develop tracheotomy complications related to hypersalivation and may develop life-threatening hyperthermia before teratoma-associated encephalitis is even suspected. Other frequent prodromal symptoms are, e.g., earache, headache, dysuria and malaise, which may be attributed to various benign etiologies. And as indicated, are also very evocative, abnormal movements of the face, mouth and tongue and symptoms of dysautonomia. Although it is not common practice to perform a gynecological examination at the time of catatonia or status epilepticus, if an X-ray, CT, MR or ultrasound demonstrates ovarian pathology or calcification, it is imperative to consider the association of ovarian teratoma and encephalitis.

### Diagnosis

Serum and CSF evaluation for anti-NMDAR antibodies and imaging tests, particularly transvaginal or transrectal ultrasound in female patients to identify small ovarian teratomas, are essential for diagnosis. In cases of brainstem cerebellar syndrome with or without teratoma-associated opsoclonus-myoclonus syndrome, anti-NMDAR antibody studies may be negative [10].

### Treatment

The combination of tumor removal and immunotherapy (ivIg, corticosteroids and plasma exchange) yields the best therapeutic results and a more rapid recovery than immunotherapy alone [4]. In the cohort study of Titulaer et al. [26] it was observed that patients without a tumor had a higher frequency of relapses than those with a tumor. Therefore, tumor resection should be performed as soon as possible after psychomotor and hemodynamic stabilization of the patient. In some cases, a delay in tumor excision has led to death [4,33], although other patients have recovered without surgery [34]. Boeck et al. [17] performed ovarectomy despite negative imaging in anti-NMDAR encephalitis. Histological examination revealed an occult teratoma and they pointed out that removal of the teratoma even 11 months after a very severe course is still therapeutically effective.

### Role of the dermoid cyst and need for its excision

Regardless of the histological type (most mature teratomas), teratomas exhibit elements of the three blastodermic layers and contain neural tissue that triggers an immune response resulting in over-production of anti-NMDAR antibodies. Dalmau et al. [35] demonstrated that all dermoid tumors examined in anti-NMDAR encephalitis patients contained neural tissue, and 100% of specimens tested were positive for NMDA receptors. Similarly, it was noted that the presence of NR2B immunoreactivity in normal human ovary oocytes may account for the occurrence of anti-NMDAR encephalitis in predominantly young females and its absence in girls [28,29]. In a case [36], a bilateral oophorectomy was performed despite the absence of a teratoma on imaging tests after the patient had failed to recover from anti-NMDA encephalitis over a 6-week period. Histology demonstrated a small, 0,7 cm mature teratoma and patient recovered afterwards. Thus, if all imaging tests are negative, it has been recommended to proceed with exploratory laparotomy and a large wedge resection of the ovary to increase the likelihood of microscopic teratoma identification and to improve the outcome in patients who are refractory to immunotherapy and have anti-NMDAR antibodies [17,30], but this issue remains controversial. We believe that a focused transvaginal or transrectal ultrasound may help decision-making better than a wedge resection, and if results are negative, all immunotherapeutic protocols should be used before proceeding to bilateral salpingo-oophorectomy that could result in ovaries macroscopically and histologically normal and menopause [37,38].

Finally, we must also remember that patients with ovarian teratoma could develop other forms of encephalitis without NMDAR antibodies and that any adolescent or young adult, particularly female, who develops subacute brainstem-cerebellar symptoms or opsoclonus-myoclonus that is suspected to be immune-mediated (because of the rapid onset of symptoms and/or CSF pleocytosis) should be investigated for teratoma of the ovary (or testes in male patients) [10].

However, the findings of Mangler et al. [39] support the current clinical practice, in which systematic screening for anti-NMDAR antibodies in teratoma patients is not indicated.

### Guidelines for future research

Heightened recognition of behavioral changes as the initial symptoms of this entity must be emphasized, followed by diagnosis through TVU and subsequent tumor removal in addition to diagnostic confirmation through the presence of anti-NMDAR antibodies. However, in some cases, the teratoma could present following the encephalitis or be microscopic. Besides, some patients with ovarian teratoma may develop other forms of encephalitis that are not anti-NMDAR-related [10] or have extra-ovarian teratomas, notably mediastinal teratomas [40,41]. These aspects should guide future investigation among encephalitis and teratoma.

## Conclusions

The association ovarian teratoma-anti-NMDAR encephalitis is a serious and potentially fatal pathology occurring in young women and under-recognized in many countries and among gynecologists.

Heightened recognition of cognitive and behavioral changes, diagnosis through TVU and subsequent tumor removal in addition to diagnostic confirmation through the presence of anti-NMDAR antibodies must be emphasized.

## Additional file

Additional file 1: Table S1. Reported cases of anti-NMDAR encephalitis and ovarian teratoma.

## Abbreviations

CT: Computarized tomography
ICU: Intensive care unit
GP: General practitioner
X-ray: Radiography
MR: Magnetic resonance
TVU: Transvaginal ultrasound
