# Supplementary material for: Ovarian teratoma-associated anti-NMDAR encephalitis: a systematic review of reported cases
Source: Orphanet J Rare Dis. 2014 Oct 14;9:157. doi: 10.1186/s13023-014-0157-x (PMC4203903; doi:10.1186/s13023-014-0157-x)
Supplement: Additional file 1: Table S1. — Reported cases of anti-NMDAR encephalitis and ovarian teratoma. [file 13023_2014_157_MOESM1_ESM.doc]

**Table S1.- Reported cases of anti-NMDAR encephalitis and ovarian teratoma.**

Abbreviations used in the table: Anti-ep, antiepileptics, anticonvulsants; AZA, azathioprine; BEP, bleomycin, etoposide, cisplatin; Chem, chemotherapy; CP, cyclophosphamide; CR, complete recovery; CS, caesarean section; ECT, electroconvulsive therapy; HA, headache; LO, left ovary; O, ovary; Ooph, Oophorectomy; PSBCh, psychiatric symptoms and behavioral changes; PSY, psychiatric symptoms; R or L S-Ooph, right or left salpingooophorectomy; R, relapse; RO, right ovary; RTX, rituximab; SIADH, syndrome of inappropriate anti-diuretic hormone secretion; TD, tumor diagnosis; TPE, therapeutic plasma exchange; TVE, transvaginal echography; (D), Dalmau.

| **Nº** | **Authors and year of pub** | **Journal** | **Country of birth** | **No. of cases with OT** | **Age/**  **(date onset)** | **Clinical presentation** | **Ovarian teratoma (T)** | **Medical treatment** | **Time to TD/Surg** | **Surgery** | **Outcome/Time to recovery** |
| --- | --- | --- | --- | --- | --- | --- | --- | --- | --- | --- | --- |
| 1 | Nokura et al, 1997 | Acta Neurol Scand | Japan | 1 | 19 | PSBCh, psychosis | Immature T  LO, 8 cm | Acyclovir+ corticoids | 28 | Tumor resection | Recovery, 4 mo, 6 mo parcial, IQ 49 |
| 2 | Okamura et al, 1997 | Lancet | Japan | 1 | 15 | August 94, PSBCh, psychosis | Immature T, RO, 15 cm | .. | 60 d | R S-Ooph,  29 sept, 94 | Recovery, 5 mo; 3 mo after surgery. CR 2,5 y |
| 3 | Aydiner et al, 1998 | J Neurooncol | Turkey | 1 | 39 | PSBCh, psychosis  1 mo after surgery | Mature T/ Immature T, RO, 6,8 cm | Acyclovir, anti-ep, Chemotherapy | 1 mo after surgery | R S-Ooph | Recovery, 12 w.  Relapse. CR 2 y |
| 4 | Taylor et al, 1999 | Can J Neurol Sci | Canada | 1 | 24 | Dermoid diagn 1 mo before, PSBCh. | Mature T, LO, 14 cm | Corticoids + IVIg | 17 d | R S-Ooph +omentectomy | Initially no relation with Derm, Recovery, 79 d. CR 6 mo. |
| 5 | Lee et al, 2003 | Acta Paediatr | Hong-Kong, China | 1 | 15 | Insomnia, HA, PSBCh, hallucinations | Immature T, LO, 20 cm + Mature T, RO, 12cm | Corticoids | ¿ | L S-Ooph + right cystectomy. | Necrosis of the hip, 6 mo. Excision of the right femoral head. Recovery at 3 y. |
| 6 | Munakata et al, 2003 | Skinkeinaika | Japan | 1 | 25 | PSBCh, psychosis, seizures | Immature T | Corticoids | - | Tumor resection | Sequelae, amnesia, seizure. |
| 7 | Fadare and Hart, 2004 | Int Semin Surg Oncol | CT, USA | 1 | 33 | MD, BCh | Mature T, RO, large | .. | 3 mo | R S-Ooph. | Recovery 1 mo, CR 1 y |
| 8 | Muni et al, 2004 | J Neuro-ophthalmol | Canada,  Chinese origin | 1 | 28 | PSBCh, psychosis.  (3 episodes) in 1998 | Recurrent: 1) Mature T, RO, 14cm 2) 2 mature T, LO, 2cm 3) Mature T, LO, 1,7cm | Corticoids + IVIg each episode. |  | 1) R S-Ooph,  2) partial L S-Ooph,  3) L S-Ooph. | Recovery (mild) at 8 w, 6 mo;  3 y after 2º episode: new surg, recovery at 6 w. 7 mo after 3º,  Recovery a 10 mo. CR 22 mo |
| 9 | Stein-Wexler et al, 2005 | Pediatr Radiol | CA, USA | 1 | 14 | PSBCh, psychosis,  Rhinorrhea, headache | Immature T, LO, 16 cm | Acyclovir, IVIg, PE, corticoids. | 10 w | L S-Ooph | Transferred to another Hospital. Died 6 mo after initial symptoms |
| 10 | Ances et al (D), 2005. | Brain | PA, USA | 1  (+ 1 med) | 26 |  | Dermoid cyst, LO, | Corticoids | 3 w | No surgery | Recovery, 14 mo |
| 11 | Vitaliani et al (D), 2005 | Ann Neurol | PA, USA | 4 | 40 | PSY, generalized seizures | Mature T, LO, 6 cm | - | 3 w | Tumor excision | 90 d of VS. Residual CD and MD at 5y |
| 12 | “ |  | PA, USA |  | 26 | PSY, generalized seizures | Dermoid cyst | Corticoids | 3 w | No surgery | 7 w of VS. CR at 14 mo |
| 13 | “ |  | PA, USA |  | 14 | Rhinorrhea, PSY, generalized seizures | Immature T | IVIg, PE | 2 mo | Surgery | Died at 6 mo |
| 14 | “ |  | PA, USA |  | 28 | 3 episodes: PSY, generalized seizures | Mature T | Corticoids, IVIg | 1 mo | Each episode: surgery | 8, 6 y 2 w of VS  Recovery at 6 mo and CR 10 mo. |
| 15 | Yang et al, 2006 | J Neurooncol | Taiwan | 1 | 35 | Convulsions, PSBCh | Mature T, RO, 20 cm, | Acyclovir, corticoids. | 6 w | R S-Ooph + bilateral cystectomy | Recovery at 3 mo. CR at 3 y |
| 16 | Lee et al, 2006 | J Ect | USA | 1 | 11 | Psychosis, probable schizophrenia, AMS | Mature T, 1600 cc cyst | ECT | 42 d | Unilateral S-Ooph | Recovery 3 mo, CR 2 y |
| 17 | Okamoto et al, 2007 | Intern Med | Japan | 1 | 35 | PSBCh | Immature T, RO. | Acyclovir, corticoids. Chem. | 4 w | R S-Ooph | Recovery, chem, CR at 2 y |
| 18 | Koide et al (D), 2007 | J Neurooncol | Japan | 1 | 19 | PSBCh | Immature T, RO, 22 cm | Corticoids. IVIg, Chem, | 4 mo | R S-Ooph. Tracheostomy | Recovery, chemotherapy |
| 19 | Dalmau et al, 2007 | Ann Neurol | USA, PA | 6 | 30 |  | Immature T, RO, 10 cm | IVIg, corticoids. | 2 mo | Tumor removal | Recovery |
| 20 | “ |  |  |  | 35 |  | Mature T, LO, 3,5 cm | IVIg, corticoids. CP | 4 mo (autopsy) | No surgery, (did not improve). | Died 4 mo after present. |
| 21 | “ |  |  |  | 25 |  | Mature T, LO, 6 cm | Corticoids. PE. | 6 w | Tumor removal | Recovery, normal at 12 mo |
| 22 | “ |  |  |  | 17 |  | Immature T, LO, 7 cm | PE, IVIg, Corticoids, CP | 4 w | Tumor removal | Recovery, normal at 7 mo |
| 23 | “ |  |  |  | 32 |  | Mature T, RO, 6 cm | Corticoids, PE. | 2 mo | Tumor removal | Recovery, normal at 6 mo |
| 24 | “ |  |  |  | 24 |  | Mature T, RO, 1,5 cm | Corticoids. | 3 mo (autopsy) | No surgery, (did not improve). | Died 3 mo after present. |
| 25 | Sansing et al (D), 2007 | Nat Clin Pract Neurol | USA, PA | 1 | 34 |  | Immature T, LO, 7,9cm | Acyclovir, Corticoids, PE, IVIg, Chem | 2-3 w? | L S-Ooph Tracheostomy. | Recovery, mild |
| 26 | Tonomura et al, 2007 | J Neurooncol | Japan | 4 | 29 | PSBCh | Bilateral mature T; 21, 6 and 4 cm | Anti-ep, Acyclovir, corticoids, IVIg | 9 mo | Bilateral ovarian cystectomy | Recovery, 21 mo |
| 27 | “ |  |  |  | 32 | PSBCh | Mature T, LO, 7.5 cm | Anti-ep, Acyclovir, corticoids, IVIg | 11 mo | Left ovarian cystectomy | Recovery, 17 mo |
| 28 | “ |  |  |  | 16 | Hallucinations, HA, BCh | Mature T, bilateral, 20 and 5 cm | Anti-ep, Acyclovir, corticoids, IVIg | 4 mo | Bilateral cystectomy | Recovery, 27 mo |
| 29 | “ |  |  |  | 17 | HA, BCh, myoclonus | Mature T, 4 cm, | Anti-ep, Acyclovir, corticoids, IVIg | 4 mo | Cystectomy | Recovery, 10 mo |
| 30 | Shimazaki et al(D), 2007 | J Neurol Neurosurg Psychiatry | Japan | 1 | 30 | PSBCh | Immature T, RO, 10 cm | Acyclovir, corticoids, IVIg | 3 mo | Tumor Resection | Recovery, 1 y. Medical resident |
| 31 | Lizuka et al (D), 2008. | Neurology | Japan | 3  (+1) | 26/  Nov 99 |  | Mature T, RO, 6 cm | Acyclovir, anti-ep. | 7 y, 3 mo | Tumor removal | Full recovery (7 y, 4 mo) |
| 32 | “ |  |  |  | 27/  Apr 00 |  | Mature T, RO, 6,6 cm | Acyclovir, anti-ep. | 6 y 10 mo | Tumor removal | Full recovery (7 y) |
| 33 | “ |  |  |  | 33/  Jan 03 |  | Mature T, RO, 4,4 cm | Acyclovir, corticoids, anti-ep | 4 y 1 mo | Tumor removal | Gradually improved over 3-4 y |
| 34 | Sabin et al, 2008 | N Engl J Med | USA, Mass | 1 | 26 | HA, PSBCh | Mature T, LO, 2,8 cm | Corticoids, IVIg, | 2 mo | L S-Ooph. | Recovery, 4-6 mo |
| 35 | Van Altena et al, 2008 | Gynecol Oncol | Netherlands | 1 | 32 | PSBCh, psychosis, seizures, | Bilateral, Inmature T, 5 cm | .. | 2 w | Laparoscopy-tomy. Bilateral S-Ooph. | Recovery. |
| 36 | Seki et al(D), 2008 | J Neurol Neurosurg Psychiatry | Japan | 1 | 18 | PSBCh | Immature T, RO, 5,8 cm | Anti-ep, Acyclovir, Corticoids. | 19 d | R S-Oph. Tracheostomy | Recovery, discharged at 129 d |
| 37 | Kataoka et al, 2008 | Eur J Neurol | Japan | 3 | 17 | PSBCh | Mature T | Corticoids, IVIg, |  | Ovarian tumor resection | Recovery |
| 38 | “ |  | Japan |  | 21 | PSBCh | Mature T | Corticoids, IVIg, |  | Ovarian tumor resection | Recovery |
| 39 | “ |  | Japan |  | 29 | PSBCh | Mature T | Corticoids, IVIg, |  | Ovarian tumor resection | Recovery |
| 40 | Kleinig et al, 2008 | Mov Disord | South Austral | 4 | 36 | PSBCh, hypersalivation | Mature T,  9 cm | Corticoids, IVIg | 5 mo | Ovarian tumor resection | Recovery, |
| 41 | “ |  | South Austral |  | 22 | PSBCh, hypersalivation | Mature T, small | Halo/droperidol | 4,5 mo | autopsy | Died, malignant catatonia.  Autopsy |
| 42 | “ |  | South Austral |  | 21 | Psychosis and catatonia | Mature T, 3 cm, LO, revealed 3 y after | ECT and neuroleptics. Corticoids. | 3 y | Ovarian tumor resection. Neural elements. | Recovery at 4 mo and discharged  At 3 y catatonia, teratoma |
| 43 | “ |  | South Austral |  | 17 | HA and BCh | Mature T, 1 cm, RO | Corticoids, IVIg, RTX. | 7 d | Ovarian tumor resection. Neural elements. | She has not yet improved 3 months later. |
| 44 | Parameswaran et al, 2008 | Neurol India | India | 1 | 15 | Intractable vomiting | Mature T, ?O | Corticoids, IVIg, | 4 w | Laparoscopic S-Ooph | Recovery, 1,5 y |
| 45 | De Bot et al, 2008 | Tijdsch v Psychiat | Holland | 1 | 32 | Psychosis, panic disorders | Immature T, bilateral | Corticoids, Chem. | 8 w | Radical, double S-Ooph | Recovery |
| 46 | Shimazaki et al(D), 2008 | Arch Neurol | Japan | 1 | 30 | HA and BCh, convulsions | Ovarian teratoma | IVIg, |  | Removal of teratoma | Recovery, at 1 y |
| 47 | Le Piane et al, 2008 | Boll Lega It Epil | Italy | 1 | 26 | PSBCh, psychiatric | Mature T, LO, | Corticoids, IVIg, anti-ep | 3 w | L Ooph. | Recovery |
| 48 | Fallica et al, 2008 | Boll Lega It Epil | Italy (from Greece) | 1 | 25 | PSBCh, psychiatric, refractory status epilepticus | Ovarian T, RO | IVIg, | Critical status | No surgery. | Recovery |
| 49 | Labate et al, 2009 | Epileptic Disord | Italy | 1 | 26 | PSBCh. | Mature T, LO, 3 cm | Anti-ep | 7 mo | Tumor removal | Recovery, 10 mo |
| 50 | Suzuki et al, 2009 | Seishin Shinkeigaku Zasshi | Japan | 1 | 22 | PSBCh. | Mature T, dermoid cyst | Psych dept  Gamma globulin | Several w | Tumor removal | Recovery |
| 51 | De Lins e Horta et al, 2009 | Eur J Gynaecol Oncol | Brazil | 1 | 32 | PSBCh, convulsions | Immature T, LO, | Acyclovir, Chem | 70 d | Radical surgery | Recovery, CR at 28 mo |
| 52 | Bayreuther et al, 2009 | Epileptic Disord | France | 1 | 25 | PSBCh, seizures, Status epilepticus | Mature T, RO | Anti-ep, Acyclovir, corticoids, IVIg. | 7 w | Tumor removal Tracheostomy, | Recovery 6 mo |
| 53 | Tillman and Raff, 2009 | JSLS, J Soc Lap Surg | USA, IN | 1 | 29 |  | Mature T, RO, 15-20 cm (focal imm.) | Acyclovir, IVIg | 2 w | Laparotomy, R S-Ooph | Recovery, 1 mo |
| 54 | Tang et al (D), 2009 | Acta Oncol | Singapore | 2 | 15 |  | 1) Immature T, LO, stage IIIA; 2) Mature T, RO | Chem: BEP | 25 d after surgical resection | Bilateral ovarian masses. Resection | Recovery, 6 w after 3 cycles of BEP |
| 55 | “ |  |  |  | 24 |  | 1) Immature T, LO, 11 cm. 2) mature T, RO | Anti-tuberculous,  IVIg, Chem: BEP | Several d | Bilateral ovarian masses. Resection | Recovery 4 mo after chemotherapy |
| 56 | Shimazaki et al (D), 2009 | BMJ Case Rep | Japan  (2004) | 1 | 30 |  | Immature T, RO, 5cm and then 10cm | Corticoids, Acyclovir, PE, IVIg | 2 mo | Considered benign, expectation, then tumor resection | Recovery.  It seems case 1 of Dalmau et al. |
| 57 | Henry et al, 2009 | Rev Neurol (Paris) | France | 1 | 35 |  | 5cm, cyst, OT containing small imm. T | Corticoids, IVIg | Several d | S-Ooph | Recovery, total at 4 mo |
| 58 | Shindo et al, 2009 | Eur Neurol | Japan | 2 | 28 | BCh, convulsions | Bilateral ovarian T, | Acyclovir, corticoids, anti-ep. | No surgery | Excellent recovery without tumor resection | Discharged at 83 d.  1 year after, anti-NMDAR not detected and bilateral teratomas without changes |
| 59 | “ |  | Japan |  | 33 | Fever and BCh | Ovarian T, RO | Anti-ep, corticoids. | No surgery | Excellent recovery without tumor resection | 5 y after, anti-NMDAR not detected and right ovarian teratoma without changes |
| 60 | Kort et al, 2009 | Obstet Gynecol | USA, NY | 1 | 20 | HA, BCh, convulsions | Mature T, LO, 1.8 cm | Anti-ep, TPE, IVIg | Gynecol consult at 5 w | Laparoscopic cystectomy of LO | Recovery, 5 mo |
| 61 | Poduval et al, 2009 | Horm Res | USA, NY | 1 | 15 | Virilization, encephalitis 16 mo before. PSBCh | Tumor 22cm, LO, mature T, Leydig cells | Carbamazepine, IVIg, | Several d | L S-Ooph | Recovery, 4 mo |
| 62 | Gable et al, 2009 | Eur J Clin Microbiol Infect Dis | USA | 2/10 | 11 | HA, BCh | Ovarian T. |  |  |  | 9 cases, recovery, 1 died.  .? |
| 63 | “ |  | USA |  | 27 | HA, psychosis | Ovarian T. |  |  |  | “ |
| 64 | Kataoka et al(D), 2009 | Mov Disord | Japan | 1 | 29 | PSBCh, seizures | Mature T. |  |  | Tumor resection | Recovery |
| 65 | De Nayer et al, 2009 | Biol Pschiatry | Belgium | 1 | 17 | PSBCh, psychiatric | Mature T, LO, 5,4 cm |  | 4,5 mo | Laparoscopic cystectomy | Recovery, 9 mo |
| 66 | Kumar et al (D), 2010 | Arch Neurol | PA, USA/ Japan | 2  (+1) | 19/  14 w of gestat. |  | Immature T, LO, 3cm, | Acyclovir, phentanyl, propofol, IVIg, corticoids. | 7 w | L Ooph.  Tracheostomy | Substantial recovery, CS at 38 w, healthy baby. |
| 67 | “ (during pregnancy) |  |  |  | 20/  8-9 w of G | History of bilateral OT removed at 16 y age. | Now bilateral mature T | Acyclovir, IVIg, corticoids.  Anti-ep. | 17 d | L S-Ooph + RO cystectomy + pregnancy terminated | Recovery.  Discharged with minimal deficits on day 87 |
| 68 | Fawcett RG, 2010 | J Clin Psychiatry | Jamaican | 1 | 28 | PSBCh, psychosis, seizures | Mature, bilateral T | Corticoids, IVIg, CP, RTX. | 2 w | Bilateral S-Ooph | Recovery, 37d, CR 1 y |
| 69 | Lizuka et al, 2010 | Brain Nerve | Japan | 1 | .. |  | Microscopic T | .. | .. | Autopsy. | Died. Teratoma detected on autopsy |
| 70 | Kleyensteuber et al, 2010 | Mil Med | USA, CA | 1 | 25 | PSBCh, Exhibition. Solic Sex ??? | Immature OT removed 2 mo earlier | IVIg, corticoids. | - | Laparotomy, Tumor removal, 2 mo before | Recovery |
| 71 | Valvat et al, 2010 | Anaesth Intensive Care | France | 1 | 41 | PSBCh, psychosis, fever, | Mature T, LO, 6.6 cm | Acyclovir, corticoids, | 3 w | L S-Ooph | Recovery 8 w |
| 72 | Berkeley et al, 2010 | Acad Emerg Med | USA | 1 | 11 | HA, BCh, psychosis | Mature T, 4,2 cm, RO | IVIg, | Several w | Resection of teratoma | Recovery, several w |
| 73 | Tachibana et al, 2010 | Intern Med | Japon | 1 | 21 | PSBCh | Mature T, RO, 5,2 cm | IVIg, corticoster, anti-ep, thiopental | 5 mo | R S-Ooph. | Recovery, several mo  (In oocytes, NMDA) |
| 74 | Lo et al, 2010 | Hong Kong Med J | Hong Kong | 1 | 21 | HA, BCh, psychosis | CT sugg T, LO, 1,3 cm. At 15 mo 3,5 cm, mature T |  | D: 2 mo  Removal: 15 mo | Removal of teratoma | Transferred other H at 4 mo  At 15 mo removal OT, Oct-2009, mild recovery, wheelchair. |
| 75 | Lesher et al, 2010 | J Pediatr Surg | USA, SC | 1 | 15 | HA, BCh, psychosis. Acute renal failure | Mature T, LO, 6 cm | IVIg, | 2 w | Laparoscopic L Ooph. | Recovery, 3-4 w |
| 76 | Sonn et al, 2010 | J Pediatr Adolesc Gynecol | USA, Mi | 1 | 14 | BCh, psychosis | Mature T, 9,5 cm, LO, | IVIg, corticoids, Trasf. Hosp, TPE | 2 w | L S-Ooph Tracheostomy, gastric tube | Discharged 3 mo after surgery. At 9 mo maintains her gastric tube. Slow recovery. |
| 77 | Le Foll et al, 2010 | Encephale | France | 1 | 24 | PSBCh, hypersalivation | Mature T, RO | IVIg, corticoids. | 3 w | Cystectomy | Recovery, 2 mo. |
| 78 | Taguchi et al, 2010 | Intern Med | Japon | 1 | 17 | BCh, psychosis, hypersalivation | Mature T, LO, | Anti-ep, corticoids, IVIg | 42 d | L S-Ooph | Hypersalivation 7 mo  Recovery |
| 79 | Prüss et al, 2010 | Neurology | Germany | 2/7 | 18 | BCh, psychosis, | Ovarian T |  |  | Tumor resection | Recovery, 50 mo.  Deficits of attention |
| 80 | “ |  | Germany |  | 19 | BCh, psychosis | Ovarian T | Corticoids |  |  | CR, 37 mo |
| 81 | Kim et al, 2010 | Korean J Pediatr | South Korea | 1 | 15 | PSBCh | Mature T, LO | IVIg. |  | Resection of tumor | Recovery |
| 82 | Xu et al, 2010 | Chinese J Neurol | China | 1 | 17 |  | Ovarian T | IVIg, corticosteroids |  | Resection of tumor | Recovery |
| 83 | Naeije et al, 2010 | Clin Neurol Neurosurg | Belgium | 1 | 29 | BCh, psychosis | Mature T, RO, 5 cm | Corticoids | 5 w | Resection of tumor | Recovery, 14 w |
| 84 | Ferioli et al(D), 2010 | Arch Neurol | USA | 1 | 26 | BCh, psychosis | Exam (-), MR: Mature T, 0,9 cm, RO | IV Corticoids | Several d | Surgical excision of T | Recovery at 5 w of surgery |
| 85 | Davies et al, 2010 | Crit Care Med | UK, Lo | 2 | 20 | BCh, psychosis | Mature T, RO, | Acyclovir, Corticoids, PE | 3 mo | Removal of teratoma | Recovery, 10 mo  A contralateral T had been removed 18 mo earlier |
| 86 | “ |  | UK, Lo |  | 21 | HA, PSBCh | Bilateral Mature T | Acyclovir, PE, Corticoids , Chem. | 5 mo | Laparoscopy, bilateral cystectomy. | Recovery, 2 y |
| 87 | Wali et al, 2011 | BMJ Case Rep | UK | 1 | 29 | Removal of dermoid 3 y ago; PSBCh. | Immature T, LO, 5cm | TPE, Chem.. | Several d | Laparoscopic L Ooph. | Gradual improvement.  Anti-NMDAR + after surg |
| 88 | Kawano et al, 2011 | Anaesthesia | Japan | 1 | 20 |  | Mature T, LO, 4 cm | IVIg | 24 d | Left ovarian tumor resection | Recovery, discharged on the 42 d postoperative |
| 89 | Taguchi et al, 2011 | Rinsho Shinkeigaku | Japan | 1 | 17 |  | Immature T, RO, | TPE, corticoids, IVIg | 27 d | R S-Ooph | Recovery |
| 90 | Challapalli et al, 2011 | North Am J Med Sci | USA, PA | 1 | 28 | Past history of right OT removed 4 y before. | Dermoid cyst, LO, 2,4cm | TPE, corticoids. | Several d | L S-Ooph | Recovery |
| 91 | Takeshita et al, 2011 | Prim Care Companion CNS Disord | Hawaii, USA | 1 | 25 |  | Mature T, LO, 4,7cm | Acyclovir, phenytoin. | 2-3 w | L S-Ooph. | Recovery |
| 92 | Estrada et al, 2011 | Patologia (Revista Latinoamericana) | Mexico | 1 | 28 | PSBCh. | Mature and Immature T, RO, 13 cm | - | 2 w | Not performed | Died at 14 d  Autopsy. |
| 93 | Caplan et al, 2011 | Psychosomatics | USA, AZ | 1 | 27 | PSBCh, psychosis | Mature T, RO, 2,6 cm | ECT, | 2 mo | Removal of the tumor?? | Recovery, 3 y |
| 94 | Millichap et al, 2011 | Pediatrics | USA, IL | 1 | 15 | PSBCh, Ictal asystole | Mature T, 2 cm | Phenobarbital, IVIg, corticoids |  | Laparoscopic partial resection of the Ov. | Recovery, 4 mo, CR 16 mo |
| 95 | Martinez-Hdez et al, 2011 | Neurology | USA, Sp | 2 | 35 | BCh, psychosis | Ovarian T |  | 4 mo | Autopsy | Died |
| 96 | “ |  | USA, Sp |  | 24 | BCh, psychosis | Ovarian T |  | 3 mo | Autopsy | Died |
| 97 | Pham et al, 2011 | J Clin Apheresis | USA, NY | 4/9 | 18 | HA, AMS | Ovarian T | Corticoids, TPE, IVIg, RTX | 10 (7-29 d) | Teratoma removal | Recovery, N/A, not available data; last time patient seen was at discharge |
| 98 | “ |  | USA, NY |  | 20 | HA |  | TPE, IVIg, RTX | 10 d | Teratoma removal | Recovery, 272 d |
| 99 | “ |  | USA, NY |  | 17 | PSY |  | IVIg, corticoids, TPE | 10 d | Teratoma removal | Did not improve in seizure control and delirium, nursing home. N/A |
| 100 | “ |  | USA, NY |  | 27 | HA, AMS |  | Corticoids, IVIg, TPE | 10 d | Teratoma removal | Limited improvement, do not resuscitate; remained nonresponsive, 272 d |
| 101 | Pascual-Ramirez et al, 2011 | Int J Gynecol Obstet | Spain | 2 | 33 | PSBCh, psychosis | Mature T, RO, hace 2 y MT in LO | Corticoids, IVIg, TPE | ws | Teratoma removal | Recovery, 1mo, (?) |
| 102 | “ |  | Spain |  | 27 | PSBCh, fever, status epilepticus | Mature T, LO, | Anti-ep, TPE, Corticoids, IVIg, | ws | Teratoma removal | Recovery, limited, (?) |
| 103 | Day et al, 2011 | J Gen Intern Med | Canada | 1/3 | 21 | PSBCh, psychosis, suicidal ideation | Mature T, RO, 2.9 cm | Acyclovir, TPE, Anti-ep, IVIg, | 3 mo | Laparoscopic right oophorectomy | Died at 14.5 w in ICU |
| 104 | Alexopoulos et al, 2011 | J Neurol | Greece | 1 | 42 | PSBCh, psychosis, fever | Mature T, LO, | Corticoids, IVIg, ICU, tracheostomy | 9 mo | CT and MR N,  At 9 mo +  Teratoma resection | Recovery 7 mo after resection, at 2 y discharged. Died in a cruise, heart attack, NMDA following + |
| 105 | Naoura et al, 2011 | Am J Obstet Gynecol | France/Mauritania | 1 | 27 | PSBCh, psychosis | Mature T, RO, 4 cm | Acyclovir Corticoids, IVIg, | 10 d | Laparoscopic cystectomy. | Recovery, 1 y attention problems remained. |
| 106 | Nakamura et al, 2011 | Seishin Shinkeigaku Zasshi | Japan | 1/2  (+1) | 17 | PSBCh, psychosis | Ovarian T | Corticoids, IVIg, | -- | Resection of teratoma | Recovery |
| 107 | Sameshima et al, 2011 | J Obstet Gynecol Res | Japan | 1 | 17 | PSBCh, psychosis | Immature T, 16 cm, RO | Corticoids, IVIg, TPE | 3 w | Laparotomy, R S-Ooph | Recovery 29 d, CR 10 mo |
| 108 | Yu et al, 2011 | Psychosomatics | Canada | 1 | 29 | 11 w postpartum PSBCh, psychosis | Immature T, RO, 3 cm | ECT, Acyclovir, Corticoids, IVIg, TPE, RTX | 2-3 w | R S-Ooph. | Recovery, discharged at 8 mo, mild recovery |
| 109 | Asai et al, 2011 | J Min Invas Gynecol | Japan | 2 | 27 | Suspected meningitis, PSBCh | Mature T, 4 cm, RO | Acyclovir, | 1 w | Laparoscopic cystectomy | Recovery in 2 w, CR 2 y |
| 110 | “ |  | Japan |  | 26 | Suspected meningitis, PSBCh. | Mature T, RO, 1 cm | Acyclovir, IVIg, | 2 mo | Laparoscopic cystectomy. | Discharged 1 mo after, Recovery, 3 mo |
| 111 | Reyes-Botero et al, 2011 | Rev Neurol | Colombia | 1 | 38 | PSBCh, psychosis | Immature T, LO, | Haloperidol, IVIg, Corticoids, Chem. | Ws? | Laparotomy, L Ooph | Recovery, 3 w, 2 mo normal |
| 112 | Uchino et al (D), 2011 | Intern Med | Japan | 1 | 21 | HA, PSBCh | Immature T, mix, 10 cm, LO | TPE, IVIg, corticoids | 3 d | L S-Ooph | Recovery, 4 mo |
| 113 | Gómez-Esteban et al, 2011 | Rev Neurol (Muñoz et al, JN) | Spain | 1 | 14 | PSBCh | Mature T, RO, 6 cm |  | 1 y after | Tumor Exeresis | Echo, CT, MR: no tumor.  Recovery; 1 y after, dermoid |
| 114 | Tanyi et al (D), 2012 | Acta Obstet Gynecol Scand | USA, PA  Spain | 3 | 34 |  | Immature T, LO, 7,9cm | TPE, IVIg, Chem | Several d | L S-Ooph | Recovery. |
| 115 | “ |  |  |  | 24 |  | Rupture, mature T, LO, 9cm | Corticoids, Emergent exploratory lap | .. | Cystectomy, Colostomy, Hartmann. | Recovery |
| 116 | Mataam et al, 2012 | Ann Fr Anesth Reanim | Belgique | 1/2 | 25 |  | Mature T, RO, | Acyclovir, tracheostomy, IVIg, | 39 d | Ovarian tumorectomy | Recovery, 18 mo |
| 117 | Dabner et al, 2012 | Int J Gynecol Pathol | Australia | 5 | 17 | Acute pain 4 mo postpartum. 20 d after surgery: PSBCh, psychosis | Immature T, RO, 9,5 cm | Corticoids, RTX. | After surgery | Laparoscopic right cystectomy. Then  R S-Ooph, radical | Recovery, 51 d ICU, 4 mo |
| 118 | “ |  | Australia |  | 37 | PSBCh, psychosis, 3 mo postpartum | Mature T, RO, 0,7 cm, histology | Corticoids, IVIg, TPE, | 6 w, after no improve. | Laparotomy, bilateral Ooph. | Recovery, 77 d ICU, discharged 100 d. Surgery for no improvement and  NMDAR + |
| 119 | “ |  | Australia |  | 27 | Seizures, PSBCh, psychosis | 1º Mature T, RO, 3,5 cm. 2º Mature T, LO, neuroglia | IVIg, and operation | 2 w,  13 mo | 1º Right ooph  2º left partial ooph | ICU 4 w,  13 mo after, again: now LO,  ICU 2 mo, recovery |
| 120 | “ |  | Australia |  | 19 | PSBCh, psychosis | Mature T, RO, 2,5 cm, neuroglia | Corticoids, IVIg, TPE, RTX | 11 d | R S-Ooph | Hospit 143 d, recovery,  Residual cognitive defect |
| 121 | “ |  | Australia |  | 17 | HA, PSBCh, psychosis, seizures | Mature T, RO, 1.8 cm, neuroglia | Corticoids, IVIg, RTX | 7 d | Laparoscopic R S-Ooph | ICU 6 mo, slow recovery  Follow deficient: enterostomy feeding problems 2 y later |
| 122 | Aoki et al, 2012 | Tokai J Exp Clin Med | Japan | 1 | 21 | HA, PSBCh, seizures, fever | Mature T, 4,4 cm, RO | Corticoids, IVIg | 26 d | R S-Ooph Tracheostomy | Recovery, discharge 105 d |
| 123 | Vural et al, 2012 | Intern Med | Turkey | 1 | 24 | PSBCh, psychosis, seizures, status epilp | Previous dermoid LO. Mature T, RO, | Corticoids, IVIg, TPE | 6-7 w | Right cystectomy | In a previous cesarean: resection of mature teratoma in LO. Recovery, several mo after 2^nd^ surgery |
| 124 | Kataoka et al, 2012 | BMJ Case rep | Japan | 1 | 17 | PSBCh, psychosis, seizures | Mature T, | Corticoids, IVIg, TPE | 5 mo | Resection cystectomy | Recovery, follow 57 mo |
| 125 | Frawley et al, 2012 | Pediatr Radiol | USA, OH | 1 | 11 | PSBCh, psychosis, seizures | Mature T, RO, 1,2 cm and 0,7 cm LO | Corticosteroids, IVIg ,TPE | 5 mo | Excision bilateral teratomas located with intraop. Echo. | Initial CT and MR interpr N, discharged at 8 w.  3 mo after, bilateral OT.  Recovery |
| 126 | Richard-Mornas et al, 2012 | Rev Med Intern | France | 1 | 41 | PSBCh | Mature T, LO | Corticoids. | 3 mo | Tumor resection | Recovery |
| 127 | Nazif et al, 2012 | Europace | USA, NY | 4 | 18 | PSBCh | Bilateral OT | Corticoids, IVIg, TPE, RTX | -- | Bilateral ovarian cystectomy | Recovery 3 mo, CR 19 m |
| 128 | “ |  |  |  | 19 | PSBCh | Ovarian T, RO | Corticoids, IVIg | -- | Right S-Ooph | Recovery 1 mo, CR 13 m |
| 129 | “ |  |  |  | 20 | PSBCh | Ovarian T, LO | IVIg, TPE, RTX | -- | Left ovarian cystectomy | Recovery 4 mo, CR 19 m |
| 130 | “ |  |  |  | 17 | PSBCh | Ovarian T, RO | Corticoids, IVIg, TPE. | -- | Right ovarian cystectomy | Recovery 2 mo, CR 5 m |
| 131 | Mehta et al, 2012 | Brit J Hosp Med | UK, Lo | 1 | 21 | PSBCh, psychosis | Mature T, | Corticoids, IVIg, TPE | 2 w | Laparoscopic resection teratoma | Remained deeply encephalopathic 4 mo after present in spite of treatment |
| 132 | Salazar et al, 2012 | Clin Neurol Neurosurg | USA, MI | 1 | 29 | PSBCh, psychosis, seizures, status ep, sialorrhea. | Mature T, LO | Corticoids, IVIg, RTX | 5 w | Laparoscopic left Ooph after NMDAR + | Echo, dermoid at Jun-10. Acute symptoms Dec-10. Oper Jan-11. Recovery 2 mo, CR 7 mo |
| 133 | Batra et al, 2012 | J Clin Apheresis | USA, NY | 1 | 34 | PSBCh, psychosis | Mature T, LO, | Acyclovir, Corticoids, IVIg, TPE | 1 w | Left S-Ooph | Recovery, 3 mo, |
| 134 | Dulcey et al, 2012 | Pathol Res Pract | Spain | 1 | 20 | PSBCh, psychosis, seizures, | Mature T, 1,3 cm, LO, necrosis | Corticoids, IVIg, CP, RTX | 3 mo | Laparotomy. L Ooph. | 1 CT negative, then MR +.  Recovery 3 mo after surg, 6 mo after initial symptoms. |
| 135 | Roberts et al, 2012 | Scott Med | UK, Sc | 1 | 33 | PSBCh, psychosis | Mature T, O? | - | 50 d | Laparoscopic Ooph | Recovery at 24 h, recovery..? |
| 136 | See et al, 2012 | J Obstet Gynaecol | UK, Eng | 1 | 31 | PSBCh, psychosis | Mature T, RO 3 cm, gliosis. | Corticoids, TPE | 56 d | After anti NMDAR +, gyn: R Ooph. | Recovery at 2 w surg  Discharged at 1 mo |
| 137 | McCarthy et al, 2012 | J Neurol | Ireland | 1 | 32 | PSBCh, during pregnancy, 1^st^ T, catatonia | Mature T, LO, 2.5 cm | Corticoids, TPE | 6 mo | CS at 32 w, finding left mass ovarian, resection. | Recovery |
| 138 | Bseikri et al, 2012 | Pediatr Infect Dis J | CA/  USA | 2/3 | 15 | PSBCh, psychosis | Ovarian T, | Corticoids, PE, IVIg, Acyclovir, CP. | 14 d | Teratoma resection + T hidden sugest. Contral. Ooph, N | Discharged 167 d  CR 1 y |
| 139 | “ |  |  |  | 11 | HA, BCh | Ovarian T | Corticoids, IVIg, | 13 | Laparoscopic Ooph. | Recovery, 55 d, CR 3 mo |
| 140 | Herrera-Julve et al, 2013 | Prog Obstet Ginecol | Spain | 1 | 17 |  | Mature T, RO, 2,3 cm | IVIg, corticoids. | 2 w | Laparoscopic cystectomy | Recovery |
| 141 | Beaudonnet et al, 2012 | La presse medicale | France | 1/2 | 24 | PSBCh, psychosis | Mature T, LO | IVIg | . | Surgical removal | Recovery |
| 142 | Nijmeijer et al, 2013 | Eur J Pediatr | Netherlands | 1 | 9 |  | Immature T, RO, 2,5cm | Acyclovir, IVIg, corticoids, TPE. | 2 w | Laparoscopic tumorectomy | Recovery, 3 mo |
| 143 | Hsu et al, 2013 | Brain Dev | Taiwan | 3 | 7 | PSBCh, seizures, SIADH | Mature T, | -- | 2 mo | Tumor removal | Recovery, CR 1 y |
| 144 | “ |  | Taiwan |  | 14 | PSBCh, seizures, hypersalivation | Mature T | Corticoids, IVIg, | 2 mo | Tumor removal | Recovery 3 mo, CR 3 y |
| 145 | “ |  | Taiwan |  | 14 | PSBCh, HA, hypersalivation | Mature T | Corticoids, IVIg, | 3 w | Tumor removal | Recovery 2 mo, recurrent 1 mo, CR 3 y |
| 146 | Boeck et al, 2013 | Case Rep Neurol Med | Germany | 1 | 34 | BCh, epileptic status  CT, FDG-PET, laparoscopy + biopsy both Ov (-) | Microscopic Mature T, RO, partial neuronal diff. | Corticoids, IVIg, PE, CP, RTX. | 11 mo | R Ooph. | Recovery 12 mo after surgery |
| 147 | Armangue et al (D), 2013 | J Pediatr | Spain | 2/20 | 17 | .. | Mature T, | Corticoids, IVIg. | -- | Unilateral Ooph | Recovery |
| 148 | “ |  | Spain |  | 13 | .. | Mature T. Follicular cyst? | Corticoids, IVIg. |  | S-Oph. | Recovery |
| 149 | Fujii et al, 2013 | Rinsho Shinkeigaku | Japan | 1 | 54 | .. | Ovarian T, RO, LE/  glioblastoma | .. | .. | Right Oophorectomy | Recovery  Glioblastoma |
| 150 | Sorita et al, 2013 | Chest | USA, NY | 1 | 35 | PSBCh, seizures, epileptic status. | Mature T, LO, 1,2 cm | Corticoids, opioids, etc. | 4 w | Left S-Ooph Tracheostomy. | ICU, prolonged resp. fail. Recovery at 1 w, 2 mo, 1 y mild. |
| 151 | Kayser et al, 2013 | JAMA Neurol | USA/Barn, Sp | 10/ 23 | 13 | PSBCh | Ovarian T | Corticoids , IVIg | - | Tumor removal | Full recovery at last, 24 mo |
| 152 | “ |  | USA/Barn, Sp |  | 18 | PSBCh | Ovarian T | Corticoids , IVIg | - | Tumor removal | Full recovery at last, 34 mo |
| 153 | “ |  | USA/Barn, Sp |  | 14 | PSBCh , relapse | Ovarian T, recurrenc at R | Corticoids , IVIg | - | Tumor removal | Full recovery at last, 35 mo |
| 154 | “ |  | USA/Barn, Sp |  | 15 | PSBCh , relapse | Ovarian T, only found at R | Corticoids , IVIg |  | Tumor removal | Full recovery at last, 47 mo  Previous screening (-) |
| 155 | “ |  | USA/Barn, Sp |  | 18 | PSBCh relapse | Ovarian T  New OT at R | Corticoids , IVIg  R: IVIg |  | Tumor removal | Full recovery at last, 13 mo  On the another ovary. |
| 156 | “ |  | USA/Barn, Sp |  | 24 | PSBCh relapse | Ovarian T | Corticoids , IVIg, RTX. |  | Tumor removal | Full recovery at last, 20 mo |
| 157 | “ |  | USA/Barn, Sp |  | 24 | PSBCh relapse | Ovarian T | PE, Corticoids , IVIg, AZA. |  | Tumor removal | Substantial >75%, 16 mo |
| 158 | “ |  | USA/Barn, Sp |  | 26 | PSBCh relapse | Ovarian T only found at R | PE, IVIg, RTX |  | Tumor removal | Substantial >75%, 126mo |
| 159 | “ |  | USA/Barn, Sp |  | 30 | PSBCh, relapse | Ovarian T only found at R | Corticoids ,RTX, CP. |  | Tumor removal | Died, pulmonary embolism , 25 mo |
| 160 | “ |  | USA/Barn, Sp |  | 34 | PSBCh, relapse | Ovarian T | Corticoids , IVIg. |  | Tumor removal | Substantial >75%, 22 mo  OT seen at initial exam but only removed at relapse |
| 161 | Dericioglu et al, 2013 | Epileptic Disord | Turkey | 1/2 | 25 | PSBCh, psychosis | Ovarian T. New OT in remaining O. | PE, Corticoids, IVIg, | 2 w | Tumor removal | Recovery 6 mo.  Previous Ooph for OT during CS |
| 162 | Jantzen et a, 2013 | BMC Neuroscience | Germany | 1 | 23 | PSBCh, psychosis | Mature T, RO, without nervous tissue | PE, Corticoids, IVIg, | 4 w,  when anti-NMDAR + | Tumor removal | Recovery 3 mo, 6 mo |
| 163 | Hacohen et al, 2013 | J Neurol Neurosurg Psychiatry | UK, Lo | 1 | 14 |  | Ovarian teratoma |  |  | ? | Case series |
| 164 | Thomas et al, 2013 (D) | JAMA Neurol | Lebanon | 1 | 30 | PSBCh | Immature T?, RO, | IVIg, Corticoids , RTX, CP, ECT. | 1.5 mo | R Ooph, Later complete L S-Ooph. | She died after a 25-month inpatient hospitalization |
| 165 | Kirchner et al, 2013 | Aktuelle Neurologie | Germany | 1 | 32 | PSBCh, psychosis | Dermoid 10 w before. 2º mature T on the another O. | Corticoids | 10 w before and 1 w after | Oophorectomy. | Recovery |
| 166 | Chia et al, 2013 | PACE | Singapore | 1/ 2 | 20 | PSBCh, seizures, rhinorrhea | Mature T, LO | IVIg | Several d, | Tumor resection | Recovery 16 d after surgery.  They present other case with Ovarian Fibroma |
| 167 | Seifi et al, 2013 | PCC, J Clin Psychiatry | USA | 1 | 19 | PSBCh, psychosis | Mature T, LO | IVIg, corticoids | 16 d | Laparoscopic L S-Ooph. | Recovery, 3 mo |
| 168 | Young et al, 2013 | Crit Care Resusc | New Zeland | 4/6w | 19 | PSBCh, ICU, pneumonia | Echo and CT N, MR: RO tumor | Corticoids, others | ? | Laparoscopic R Ooph. | ICU 45 d; hospital stay 145 d.  Ovarian teratoma |
| 169 | “ |  | New zeland |  | 28 | PSBCh, fever, seizures | TVU: Right adnex mass, Ovarian T. | Corticoids, others | ? | Laparoscopic bilateral partial Ooph. | ICU 35 d; hospital stay 58 d  Recovery.  Only right ovarian teratoma |
| 170 | “ |  | New zeland |  | 23 | PSBCh, HA, epileptic status | TVE: Bilateral ovarian T. | Corticoids, others | ? | Laparoscopic bilateral Ooph, | ICU 82 d; Remains in hospital after 2 y.  Bilateral teratomas |
| 171 | “ |  | New zeland |  | 38 | HA, photophobias, myalgias, PSBCh. | Echo and RM: N  Unilateral T | Corticoids, others | ¿ | Laparoscopic bilateral Ooph, | ICU 79 d; hospital stay 110 d, Recovery.  Unilateral Teratoma |
| 172 | Reid and Clardy, 2013 | J Neurol Neurosurg Psychiatry | USA | 1 | 19 | PSBCh, | Mature T, RO, 1,9 cm | PE | 1 w | Removal teratoma | Recovery |
| 173 | Maraka et al, 2013 | Connect Med | USA | 1 | 33 | PSBCh, seizures | Mature T, RO, 1,4 cm | Acyclovir, IVIg, corticoids. | 15 d | R S Ooph. Tracheostomy, | Recovery, transferred to other Hospital at 21 d |
| 174 | Acién et al (2014) | J Obstet Gynaecol | Spain | 1 | 24 | HA, otalgia, PSBCh, fever | Mature T, RO, 0,8 cm | Acyclovir, IVIg, corticoids. Anti-ep. | 20 d | R Ooph. | Recovery 1,5 mo; CR 1 y  OT and LE + Myoclonus |
